# Total Elbow Arthroplasty as the Treatment of Choice for a Young Man with Neglected Terrible Triad of the Elbow Joint and Schizophrenia: A Case Report

**DOI:** 10.5704/MOJ.2411.009

**Published:** 2024-11

**Authors:** SD Savio, KYW Artha, IGLNAA Wiguna

**Affiliations:** Department of Orthopaedics and Traumatology, Udayana University, Denpasar, Indonesia

**Keywords:** case report, schizophrenia, terrible triad, total elbow arthroplasty

## Abstract

In young patients, the use of total elbow arthroplasty (TEA) is rarely preferred due to its high rate of mechanical failure. Poor compliance and psychological problems encountered may lead to increased difficulty in management. A 38-year-old male complained stiffness and pain on his left elbow. History of trauma was present 10 months ago, when he fell down from a tree of 6m high. Immediate closed reduction and immobilisation with backslab was performed, but he was lost to follow-up due to Schizophrenia. In physical examination, we found varus and recurvatum deformity with inability to flex the elbow beyond 30° and perform pronation. Plain radiograph and CT scan confirmed the terrible triad of elbow with callus formation. Total elbow arthroplasty with soft tissue release was then performed, resulting in satisfactory range of motion at one year follow-up. The management of neglected terrible triad of the elbow is challenging not only due to the bony problems, but also contracted muscles and fibrotic joint. TEA previously has been described in cases of inflammatory arthritis and degenerative arthritis, less in post-traumatic conditions especially in young patients. Though there is still scarcity in literatures discussing the burden of psychiatric problems in arthroplasty patients, but the existing literatures proved the correlation between psychiatric comorbidity with higher rate of post-operative adverse events. Total elbow arthroplasty can be considered as a surgical treatment for a young patient with neglected fracture dislocation of elbow with satisfactory result; however close post-operative monitoring and routine physiotherapy exercise should always be performed.

## Introduction

Even though commonly seen in daily practice, fracture dislocation of the elbow joint is often underestimated by the patients due to lack of health awareness in developing countries. Neglected fracture dislocation of the elbow joint, defined as untreated pathology for three weeks or more, presents as a challenge to surgeons, due to soft tissue contractures and uncertain outcomes. Treatment options range from open reduction, external fixator, ligament reconstruction, up to elbow arthroplasty.

Although its efficacy is established for degenerative pathologies in elderly, the use of Total Elbow Arthroplasty (TEA) is rarely preferred in young patients due to its high rate of mechanical failure. A study by Schoch *et al* (2017) stated that TEA in young population less than 50 years old should be approached with cautious, as the failure rate of TEA in young patients could reach up to 82%, with most of them requiring additional medical procedures due to implant loosening, infections, stiffness, or muscle weakness^1^.

Poor compliance and psychological problems encountered during the treatment may also lead to increased difficulty in managing neglected fracture dislocation of the elbow. However, until now, there has been limited literatures on the effect of psychiatric comorbidities in patients undergoing arthroplasty. Through this case report, we aim to describe the challenge encountered in managing a young patient with a history of schizophrenia and old unreduced fracture dislocation of the elbow joint, up to one year of follow-up.

## Case Report

A 38-year-old male came to our outpatient clinic with stiffness and pain on his left elbow. The pain was continuous, disturbing his daily activity and sleep. He also complained that he could not flex his elbow, and when he extended it, it would become hyperextended. Tingling, hand grip weakness, and numbness was denied. History of trauma was present 10 months ago, when he fell down from a tree of 6m high with his left elbow bumped to the ground. Plain radiograph in emergency room demonstrated the terrible triad of the left elbow, with posterior elbow dislocation, coronoid and radial head fracture. Immediate closed reduction and immobilisation with backslab was performed under general anaesthesia, and he was then planned for subsequent reconstructive surgery. However, he was then lost to follow-up as he was diagnosed with Schizophrenia, resulting in him removing the backslab and ceasing routine control. As his psychiatric condition improved under medication, his family took him back to the orthopaedic clinic to continue the treatment. During the 10 months lost to follow-up, he was only given mild analgetics (Paracetamol 500mg) when he complained of pain. At the time, he regularly consumed Risperidone 2mg daily per oral from the psychiatrist and had been calm and co-operative during the whole outpatient and inpatient treatment.

**Fig. 1:**
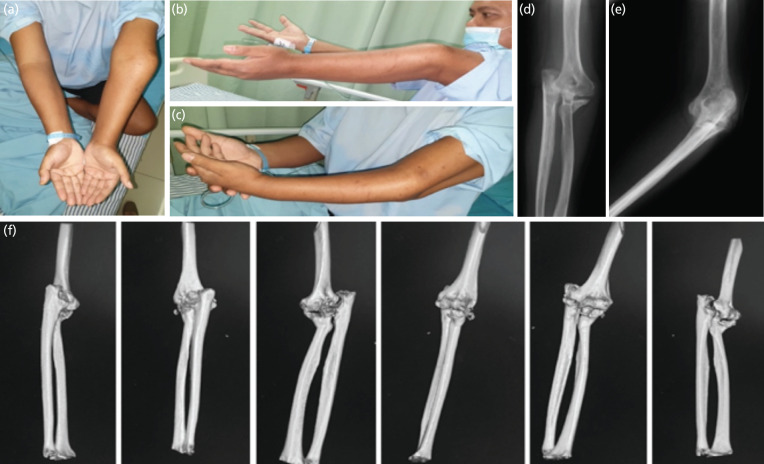
(a, b, c) Pre-operative clinical pictures, (d, e) Plain radiographs, and (f) CT Scan result of the patient.

In physical examination, we found varus and recurvatum deformity with inability to flex the elbow beyond 30°. Active supination range of motion was 90° and pronation range of motion was 0°. Pre-operative clinical presentation was described in (Fig. 1 a-c). Plain radiograph in anteroposterior (Fig. 1d) and lateral view (Fig. 1e) showed posterior dislocation of elbow joint, coronoid fracture with radial head fracture with marked displacement and callus formation. CT scan confirmed the extent and comminution of fractures (Fig. 1f), and total elbow arthroplasty (TEA) with soft tissue release was the treatment of choice for this case.

**Fig. 2:**
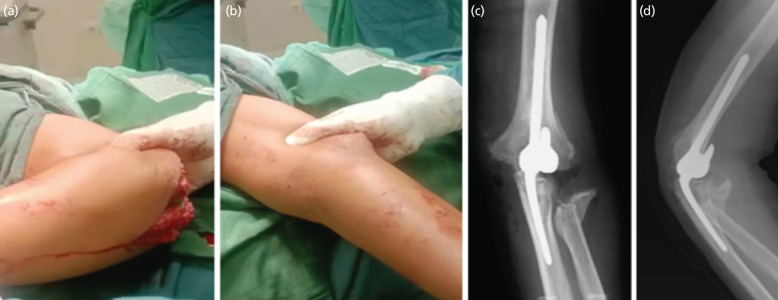
(a, b) Intra-operative image showing passive ROM of 0/100. (c) Post-operative radiograph in AP, and (d) lateral view.

Intra-operatively, the patient was positioned in lateral decubitus, and posterior approach with triceps splitting was performed on his left elbow. The fracture configuration was difficult to identify as there was abundant fibrous tissue and callus formation. Careful but extensive soft tissue release was performed, and ulnar nerve was preserved. Radial head was excised due to the unreconstructible radial head condition, then humeral and olecranon preparation was performed. The prosthesis was installed [Total Elbow System 100mm x 75mm, Symmex, Indonesia], and intraoperative passive flexion range of motion reached up to 1000 while the extension range of motion was up to 0° (Fig. 2 a-b). Triceps lengthening was performed using V-Y technique to address triceps contracture. Final post-operative plain radiographs were shown in (Fig. 2 c-d).

**Fig. 3:**
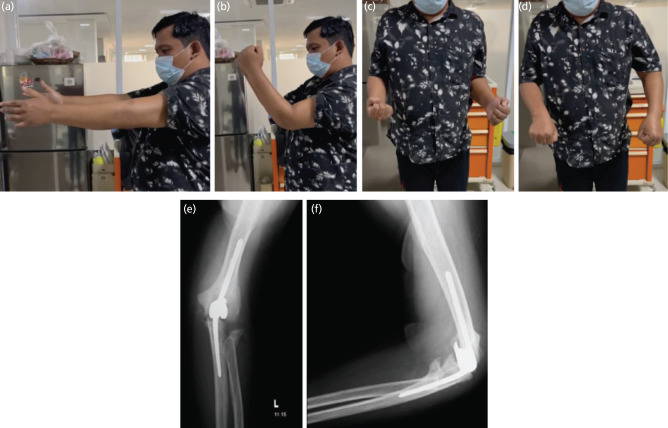
(a, b, c, d) Clinical condition one year post-operatively and radiograph evaluation showing implant in good condition with no sign of loosening, (e) in AP and (f) lateral view.

Post-operatively, antibiotics and analgetic was given along with regular wound care every 2-3 days. Active range of motion was encouraged in collaboration with physiotherapist in our hospital. One year post-operatively, the post-op scar showed no signs of dehiscence nor nerve lesions. His left elbow was painless, and the range of motion evaluation showed extension of 0°, flexion of 75°, supination of 45°, and pronation of 45° (Fig. 3a-d) with full motoric power of all movements. The disabilities of the arm, shoulder, and hand (DASH) score was 7.5% showing satisfactory functional outcome. No complication was found during the one-year follow-up period. Evaluation of plain radiographs in anteroposterior (Fig. 3e) and lateral view (Fig. 3f) one-year post-operatively showed that the implants are in good position with no signs of loosening. The patient’s mental health was also in stable condition under medication, and he could perform daily activities independently.

## Discussion

Total Elbow Arthroplasty (TEA) previously has been described in cases of inflammatory arthritis and degenerative arthritis, but less in posttraumatic conditions especially in young patients. According to a study by Celli *et al*, 2009, TEA was only performed in 8% of patients less than 40 years old, and posttraumatic conditions were indicated only in 35% of patients while inflammatory conditions were found in 65% of patients. In young patients with elbow arthritis, arthroplasty is more suitable when extensive bone loss, gross instability, or fixed deformity is observed. Other treatment methods for such conditions are capsulectomy, resection arthroplasty, arthrodesis, interposition arthroplasty, and hinged-bar external fixator^2^.

The management of neglected terrible triad of the elbow is challenging not only due to the bony problems, but also contracted muscles and fibrotic joint, collateral ligament contracture, and possible nerve involvement. There have also been controversies regarding the use of linked or unlinked elbow arthroplasty. In this patient, we used linked TEA, as unlinked arthroplasty requires competent medial collateral ligament and linked arthroplasty is more suitable when significant bone loss exists. A systematic review by Davey *et al* (2021) proved that the infection and revision rate do not vary between linked and unlinked prosthesis; however linked devices are superior in terms of lower dislocation rate and unlinked devices have been related to lower incidence of nerve injury. Aseptic loosening was thought as the most frequent cause of revision, and it was proven that linked devices resulted in lower rate of aseptic loosening. On the other hand, unlinked arthroplasty tends to have more instability and dislocations as the cause of revision surgeries^3^. Tight triceps in our patient was dealt using V-Y lengthening. As this procedure might weaken the triceps mechanism and extensive surgical trauma might result in heterotopic ossification, we tried to perform this procedure as carefully as possible to maintain the integrity of triceps mechanism in order to maximise the clinical result postoperatively.

Post-operative rehabilitation also plays important role in the surgical success. Besides regular post-operative care such as limb elevation and active finger movements, for patients treated with linked arthroplasties like our case, the elbow can be put on rest using arm sling or splint in 90° flexion position for at least 6 weeks. Active assisted elbow flexion can be started 24 - 48 hours after surgery, followed with gravity assisted extension to protect triceps repair for 6 weeks. Lifetime restrictions for these patients are lifting objects more than 5kg weight and upper extremity impact sports. Failure to follow proper post-operative care could lead to complications. Some complications observed in previous literatures needing additional surgical procedures are infections, loosening (especially ulnar component), mechanical failure, and triceps insufficiency^2^. Being one of the most common problems found in young population, mechanical failure can be caused by excessive elbow practice and non-adherence to follow post-operative restrictions^1^.

Though there is still scarcity in literatures discussing the burden of psychiatric problems in arthroplasty patients (especially upper limb arthroplasty), but the existing literatures proved the correlation between psychiatric comorbidity and higher rate of post-operative adverse events. Patients with depression and schizophrenia tend to have higher rate of blood transfusion perioperatively. Anaemia was also often misdiagnosed in these patients as the symptoms of fatigue can mimic anaemia. Lower compliance is another problem in this population, as it contributes to worse outcomes. A more recent study by Kheir *et al* (2018) in patients with total hip/knee arthroplasty and schizophrenia/bipolar disorders proved that these patients have higher rate of periprosthetic joint infection, due to defective immune status caused by chronic psychiatric disorders leading to increased level of inflammatory cytokines such as interleukin-6, tumour necrosis factor alpha, and interferon gamma. Implant loosening and dislocation were also more commonly found in these patients, due to possible impaired perception, lack of insights, hygiene neglect, and self-harming behaviours^4^. Another case report by Barati *et al* (2023) described a young patient with bilateral open terrible triad injuries and a history of psychoactive substance abuse. The patient was treated with antibiotic-loaded radial head spacer arthroplasty on one side and open reduction with internal fixation on the other. Metallic prostheses were contraindicated due to poor skin condition. Post-operatively, the patient was lost to follow-up due to his psychiatric condition, resulting in a significantly restricted range of motion in the elbows at the six-month follow-up^5^.

In contrast, our patient received acute definitive treatment and maintained a stable psychiatric condition during follow-up, which is crucial for recovery. The first month’s post-surgery are critical for functional improvements, highlighting the need to stabilise chronic illnesses or psychiatric conditions before definitive surgical procedures.

This study has several limitations. Firstly, it is a case report of a single patient, providing Level IV evidence and limiting the generalisability of the findings. However, this study still serves as an important lesson for orthopaedic surgeons when dealing with complex cases that involve psychiatric conditions. The follow-up period was also restricted to one year, as the patient could not be contacted afterward. Although it may not be sufficient to fully assess long-term results and late-onset complications, a one-year follow-up still offers valuable insights into the initial outcomes and early complications of these kinds of treatment.

Even though some literatures have shown the efficacy of TEA in post-traumatic elbow in young patients, but this treatment should always be approached cautiously as there is still risks of complications. In addition, early mobilisation training, proper wound care, and close monitoring play’s important role in treatment success. In patients with psychiatric issues, social support should always be maintained to ensure maximal outcome.
